# Network analysis of human post-mortem microarrays reveals novel genes, microRNAs, and mechanistic scenarios of potential importance in fighting huntington's disease

**DOI:** 10.1016/j.csbj.2016.02.001

**Published:** 2016-02-10

**Authors:** Sreedevi Chandrasekaran, Danail Bonchev

**Affiliations:** Center for the Study of Biological Complexity, Virginia Commonwealth University, Richmond, VA, USA

**Keywords:** Huntington's disease, Computational molecular neurobiology, Transcriptome, Protein interaction network, miRNAs, Microarray analysis

## Abstract

Huntington's disease is a progressive neurodegenerative disorder characterized by motor disturbances, cognitive decline, and neuropsychiatric symptoms. In this study, we utilized network-based analysis in an attempt to explore and understand the underlying molecular mechanism and to identify critical molecular players of this disease condition. Using human post-mortem microarrays from three brain regions (cerebellum, frontal cortex and caudate nucleus) we selected in a four-step procedure a seed set of highly modulated genes. Several protein–protein interaction networks, as well as microRNA–mRNA networks were constructed for these gene sets with the Elsevier Pathway Studio software and its associated ResNet database. We applied a gene prioritizing procedure based on vital network topological measures, such as high node connectivity and centrality. Adding to these criteria the guilt-by-association rule and exploring their innate biomolecular functions, we propose 19 novel genes from the analyzed microarrays, from which *CEBPA*, *CDK*1, *CX3CL1*, *EGR1*, *E2F1*, *ERBB2*, *LRP1*, *HSP90AA*1 and *ZNF148* might be of particular interest for experimental validation. A possibility is discussed for dual-level gene regulation by both transcription factors and microRNAs in Huntington's disease mechanism. We propose several possible scenarios for experimental studies initiated via the extra-cellular ligands TGFB1, FGF2 and TNF aiming at restoring the cellular homeostasis in Huntington's disease.

## 1 Introduction

In 1872, a young American physician named George Huntington was the first to recognize a specific inherited neurodegenerative disorder. Later it was named after him as Huntington's disease (HD) and in early 1990s the mutant gene Huntingtin (HTT) was discovered to be the cause of the disease. Expansion of 36 or more CAG trinucleotide (polyQ) repeats in HTT gene is the hallmark characteristic of the disease. PolyQ expanded HTT is considered as a trigger of the neurodegeneration that eventually caused all the Huntington's disease symptoms. The disease is described by progressive motor, cognitive as well as emotional disturbances. The motor symptoms include chorea, dystonia, rigidity, postural instability, etc. Depression and personality changes are the major emotional disturbances part of the disorder. Like Alzheimer's disease (AD), short-term memory loss, confusion and disorientation are some of the cognitive issues found in HD patients.

**Fig. 1:**
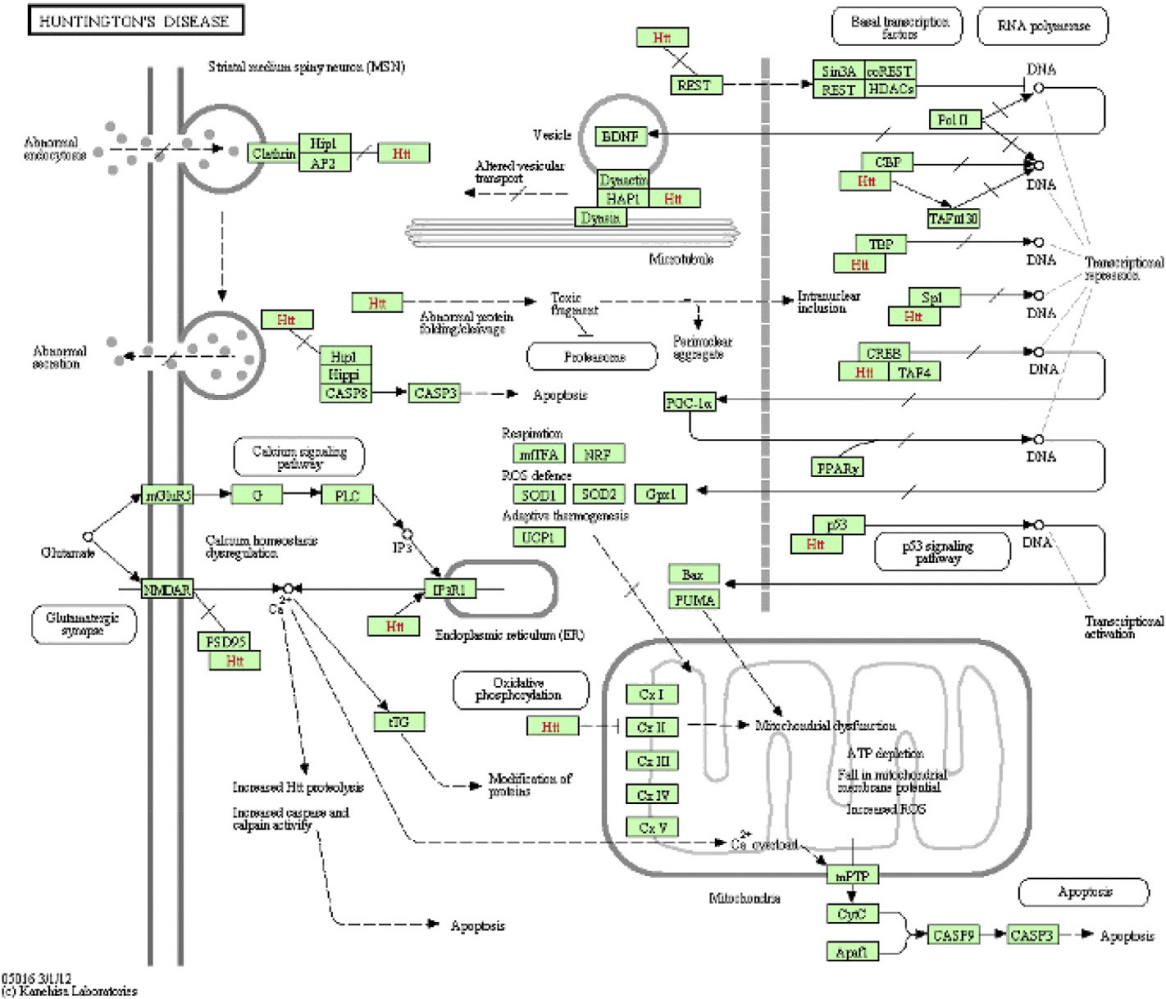
**Huntington's disease pathway from KEGG database.** Biological processes and genes implicated in the Huntington's disease. Courtesy: Huntington's disease pathway from KEGG database, available at http://www.genome.jp/kegg/pathway/hsa/hsa05016.html retrieved on Apr. 3, 2013.

In Huntington's disease, it was suspected that the neurodegeneration is selective for striatal GABAergic medium-sized spiny neurons. These neurons project to substantia nigra and globus pallidus parts of the brain affecting primarily motor coordination [1], [2], [3]. Fig. 1 depicts HTT gene along with its interacting partners which trigger the striatal neuronal loss that eventually manifests all the Huntington's disease symptoms. Like the other neurodegenerative disorders Parkinson's disease and Alzheimer's disease, HD also showed protein misfolding, ubiquitin proteasome system deregulation, autophagy dysfunction, metabolic and mitochondrial dysfunction as well as oxidative stress, which over the years culminates into motor and cognitive disorders [4], [5], [6], [7]. The advances in understanding the molecular pathogenesis of HD, and the multiple research approaches undertaken since the discovery of the HD gene in 1993, are reviewed by Zuccato et al. [8]. Mattson summarized the evidence for the role of apoptosis and related pathways of oxidative stress, perturbed calcium homeostasis, mitochondrial dysfunction and caspases activation in neurodegenerative diseases. His analysis involved the protecting survival signals, which suppress oxygen radicals and stabilize calcium homeostasis and mitochondrial function [9].

In addition to HTT, mutations in HDL3, JPH3 and PRNP genes were also related to Huntington's disease pathogenesis (OMIM database, retrieved on Dec. 17, 2012). Other genes such as CCKBR, cytochrome c and GAPDH were known to contribute to the diseased state. It was reported that there is a possible selective loss of Cholecystokinin receptors (CCKBR) containing neurons in cerebral cortex of Huntington's patients [10]. Cytochrome c release from mitochondria triggers the downstream caspase activation leading to apoptotic neuronal death in many neurodegenerative diseases. This kind of neuronal death plays a greater role at the end stage of HD [11]. Due to its selective binding to the CAG repeats in huntingtin gene, GAPDH activity was found reduced in HD brains, thereby reducing the cellular energy production [12], [13].

The role of FOXO gene for preserving the normal neuronal capacity, a role that is suppressed by the Wnt receptor Ryk in HD was studied by Tourette et al. [14]. Such models also helped to identify genes that regulate the dysfunction of mutant polyglutamine neurons [15]. Brain-derived neurotrophic factor (BDNF) has been suggested to reduce amyloid-β neurotoxicity in Alzheimer's disease [16], [17], [18]. Some of the suggested beneficial pathways up-regulate the innate autophagy process and via increasing BDNF gene expression. Autophagy process protects against the toxic insults of mutant huntingtin proteins by enhancing its clearance from the cell. BDNF is necessary for the survival of striatal neurons in the brain and it promotes synaptic plasticity in addition to memory formation. It can also act as a neuromodulator affecting the pre-synaptic release of neurotransmitters in central nervous system. Along with BDNF, genes like BCL2 and G-protein coupled receptors (GPCRs) could also be part of therapeutic measures in Huntington's disease. Human antioxidant defense proteins that were strongly induced in striatum, but also detectable in cortex, were identified as peroxiredoxins 1, 2, and 6, as well as glutathione peroxidases 1 and 6 [19].

Huntington's disease research benefited considerably from animal models (*C. elegans* and particularly mouse). These models expand the knowledge base of the disease, act as a valuable experimental tool for preclinical therapeutic trials, and provide biomarkers of disease progression which could be detectable in skin, muscle, blood or other peripherally accessible tissue [20], [21]. This resulted in few valuable therapeutic measures to alleviate the disease symptoms. HD animal model data suggest a possible interaction between genetic and environmental factors [22]. Novel transcriptional changes in several genes that were involved in synaptic integrity and function were found in HD mice [23]. Another HD animal model study demonstrated that the mutant huntingtin directly or indirectly reduces the expression of a distinct set of genes involved in signaling pathways that were known to be critical for the functioning of striatal neurons [24]. When administered systemically or delivered via genetically-grafted cells, BDNF has shown to prevent striatal neurons from cell death in HD animal models [25]. FGF2 (fibroblast growth factor 2) was shown to improve motor performance and extend the lifespan by 20% by reducing the accumulation of polyQ aggregates in the brain [26], [27]. Zhang et al. [28] found that mouse caspase activation precedes pro-apoptotic changes in Bcl-2 family members. Understanding the chronology of apoptotic events provides important information for appropriate therapeutic targeting in this devastating and untreatable disease. Decreasing IRS2 (insulin receptor substrate 2) signaling could be part of a therapeutic approach to slow down the progression of HD [29]. Thier abundant presence in central nervous system as well as their complex interactions with many downstream targets have made GPCRs potential drug targets in many neurological diseases including Huntington's disease [30]. The transcription factor XBP1 deficiency was found to lead in animal studies to augmented expression of FOXO1, a key transcription factor regulating autophagy in neurons [31].

Recently, three papers used one of the two microarray datasets analyzed in our study (GSE3790 with Affymetrix GeneChip Human Genome HG-U133A). Neueder and Bates [32] uncovered previously unidentified transcription dysregulation in the HD cerebellum, and found that genes implicated in mitochondrial function, glycolysis, intracellular protein transport, proteasome and synaptic vesicles are commonly negatively correlated with HD in the cerebellum, frontal and caudate networks. Studying molecular mechanisms of HD Kalathur et al. [33] found indications for potential relevance of the cell cycle processes, RNA splicing, Wnt and ErbB signaling, and proposed a candidate set of 24 novel genetic modifiers. Alcaraz et al. [34] used the GSE3790 dataset in one of the case studies to prove the efficiency of their KeyPathwayMiner computational tool. An interesting moment in their analysis is that some of the proposed new HD-relevant genes (termed “exception” genes) are statistically insignificant. This approach (although not rigorously defined) parallels one part of the strategy of the present authors used in preceding articles [35], [36], [37] under the name “connecting” proteins (vide infra).

Since its discovery, a multitude of genetic and biomolecular research studies have contributed valuable information about the Huntington's disease underlying mechanism and the critical genes that were deregulated in the process. In this research work, we have utilized extensive network techniques to further expand the knowledge base of the Huntington's disease's biological mechanisms and the vital molecular players. Network-based analysis is a useful tool to understand and appreciate the underlying complexity of a system as a whole rather than a disconnected unit. This research work on Huntington's disease is part of a comprehensive study to understand the underlying common molecular mechanisms and genes involved in neurodegenerative diseases including Parkinson's disease and Alzheimer's disease. Our more important network-based findings about the latter two diseases can be found in Refs. [36], [37].

## 2 Material and methods

The study rationale and workflow are similar for all three neurodegenerative disorders (NDDs) (Parkinson's, Alzheimer's and Huntington's diseases) that we chose to study in understanding the underlying common biomolecular mechanisms and genes. Detailed information about the study methodology is presented in our Parkinson's disease journal article [36]. Below, we describe the various steps involved in network-based analysis of Huntington's disease.

Microarray gene expression data is the fundamental means to carry out network-based analysis. Such datasets are available in major public data repositories like National Center of Biotechnology Information's (NCBI) Gene Expression Omnibus (GEO) and European Bioinformatics Institute's (EBI) ArrayExpress databases. For uniformity and cross-validation, Affymetrix microarray gene expression datasets were used for all three NDDs. For Huntington's disease, NCBI GEO's GSE3790 dataset [38] which contained post-mortem human brain tissue samples from both HD patients and controls (44 and 36 samples, respectively) taken from three brain regions namely cerebellum, frontal cortex and caudate nucleus was used. For a detailed information about the samples, refer to Refs. [38], [39]. The microarray expression dataset was then normalized using the Robust Multi-array Average (RMA) approach [40]. The differential gene expression changes were statistically evaluated by the empirical Bayes (eBayes) method from the limma Bioconductor package [41], [42]. Probe-sets with *p*-values of < 0.05 were considered to be significantly differentially expressed genes (SDEGs).

The GSE3790 microarray gene dataset had both Affymetrix GeneChip Human Genome HG-U133A and B. Following the above mentioned statistical plan, the two datasets were analyzed and the significantly differentially expressed genes lists called “seed genes” were generated. The lists generated from the GSE3790 dataset were denoted as CE, FL and CN, for the three types of brain tissue samples: cerebellum, frontal cortex and caudate nucleus, respectively. In addition, a “diagnosis” differential genes list was generated utilizing *all* the 201 microarray samples in which there were 87 controls and 114 HD cases. By including the diagnosis set, we were able to filter our SDEGs in a little more stringent and concise way.

**Fig. 2:**
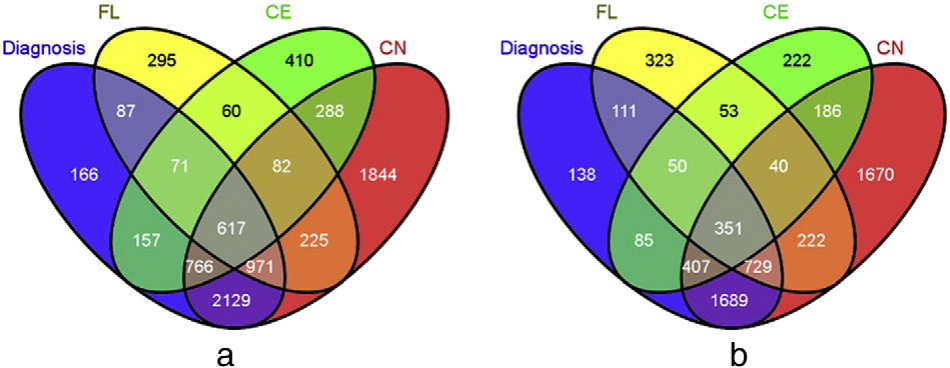
(a) and (b). Four-set Venn diagram of the overlap of significantly differentially expressed genes (SDEGs) in (a) GSE3790 HG-U133A and (b) GSE3790 HG-U133B gene expression datasets. Courtesy: Oliveros, J.C. (2007–2015) Venny. An interactive tool for comparing lists with Venn's diagrams. http://bioinfogp.cnb.csic.es/tools/venny/index.html.

A four-step strategy was applied for selection of “seed” genes in our analysis. It starts with the strongly modulated genes in each of the three types of brain tissues, then identifying the overlapping genes between different tissues from the *same microarray*, next eliminating the redundant genes from the overlap between the selected sets of the *two microarrays*. In GSE3790 HG-U133A, an overlap of 617 seed genes was found between the four sets of significantly differentially expressed genes (SDEGs), as shown in Fig. 2a. In a similar way, an overlap of 351 seed genes was found in the GSE3790 HG-U133B microarray gene expression dataset (Fig. 2b). Altogether, 925 seed genes were found after removing duplicates in GSE3790 U133A and B datasets. In order to partially compensate for not accounting for the multiple correlation, and still have a considerable number of overlapping genes, the fourth selection step included in the network evaluation only those SDEGs with the *p*-value lower than 0.01. Following this new cut-off criteria, 531 genes were treated as “seed genes” which were then subjected to comprehensive network analysis (See Supplementary Table S1 for the list of 531 SDEGs).

Pathway Studio 9.0 software package (http://www.elsevier.com/online-tools/pathway-studio) along with its proprietary molecular interaction database namely ResNet 9.0 (released October 15, 2011) was utilized to construct various networks such as *direct* interaction, *shortest-path* and *miRNA* regulation [43]. In addition, critical network topological characteristics such as node degree (local connectivity), closeness centrality (network monitoring) and betweenness centrality (traffic-influential) scores were also calculated using the Pajek software package [44], [45]. Node degree is defined through the number of nearest neighbors in the network. The larger this number, the stronger the influence of this network node on its neighborhood in case of positive or negative modulation. Closeness centrality is defined as reciprocal of the sum of distances from a node to all other nodes in the network. High closeness centrality means more effective monitoring the network from a given node. Betweenness centrality measures another aspect of central location—the possibility to influence a larger portion of the network node–node communications. In short, these three measures of local, respectively global connectivity and centrality determine the speed with which harmful or/and beneficial signals will be transmitted through the entire system. Based on these prioritizing topological characteristics, as well as on their biological/molecular functions relevant for the neurodegenerative process, the seed genes were categorized as “already known HD-genes” and “genes of interest for HD”. Such categorization was possible after careful review of various sources like Online Mendelian Inheritance in Man (OMIM) database (http://omim.org/), NCBI's PubMed database (http://www.ncbi.nlm.nih.gov/pubmed.com), MalaCards database (http://malacards.org/), and Google search for the latest publications (http://www.google.com). Later, the two categories were further divided in two subcategories, those found among the significantly differentially expression genes (SDEGs) and such emerging from the connecting proteins in *shortest-path* network.

Further the seed genes were subjected to Gene Ontology (GO) enrichment analysis using Database for Annotation, Visualization and Integrated Discovery (DAVID), a widely used Web-based application focusing on GO classification [46], [47], [48]. GO enrichment analysis provides biological functional interpretation of large lists of genes derived from genomic studies such as microarray, proteomics experiments, etc. We also used the core analysis in Ingenuity's IPA (Ingenuity Systems, http://www.ingenuity.com/) and Pathway Enrichment Analysis in Pathway Studio to explore the various canonical pathways that could be affected in Huntington's disease.

In conclusion, an *integrated* mechanistic disease network was constructed using the genes/proteins found in common in all enriched Kyoto Encyclopedia of Genes and Genomes (KEGG) pathways resulted from DAVID analysis [49]. Again, Pathway Studio software was used to construct the *direct interaction* network using the “mechanism genes” (genes found in common in all enriched KEGG pathways) to investigate the *integrated* Huntington's disease mechanism.

## 3 Results and discussion

Using the GSE3790 dataset, the original authors had performed gene set enrichment analysis using DAVID tools to identify biological processes and pathways significantly affected in HD [39]. One of the major conclusions of this study was that the differential gene expression in HD brains showed distinct regional pattern similar to an already known pattern of neuronal loss. The greatest number and magnitude of differentially expressed genes/proteins were detected in the caudate nucleus, followed by motor cortex, then in cerebellum tissue types.

Our statistical analysis with renormalized data revealed a similar gene expression difference in these neuronal tissue samples. In this study, we expanded upon their research work by subjecting the differentially expressed genes to various network techniques to explore the underlying cellular mechanisms and molecular players of Huntington's disease. We initiated our HD network analysis using 531 “seed genes”.

### 3.1 Huntington's disease *direct* interaction network

**Fig. 3:**
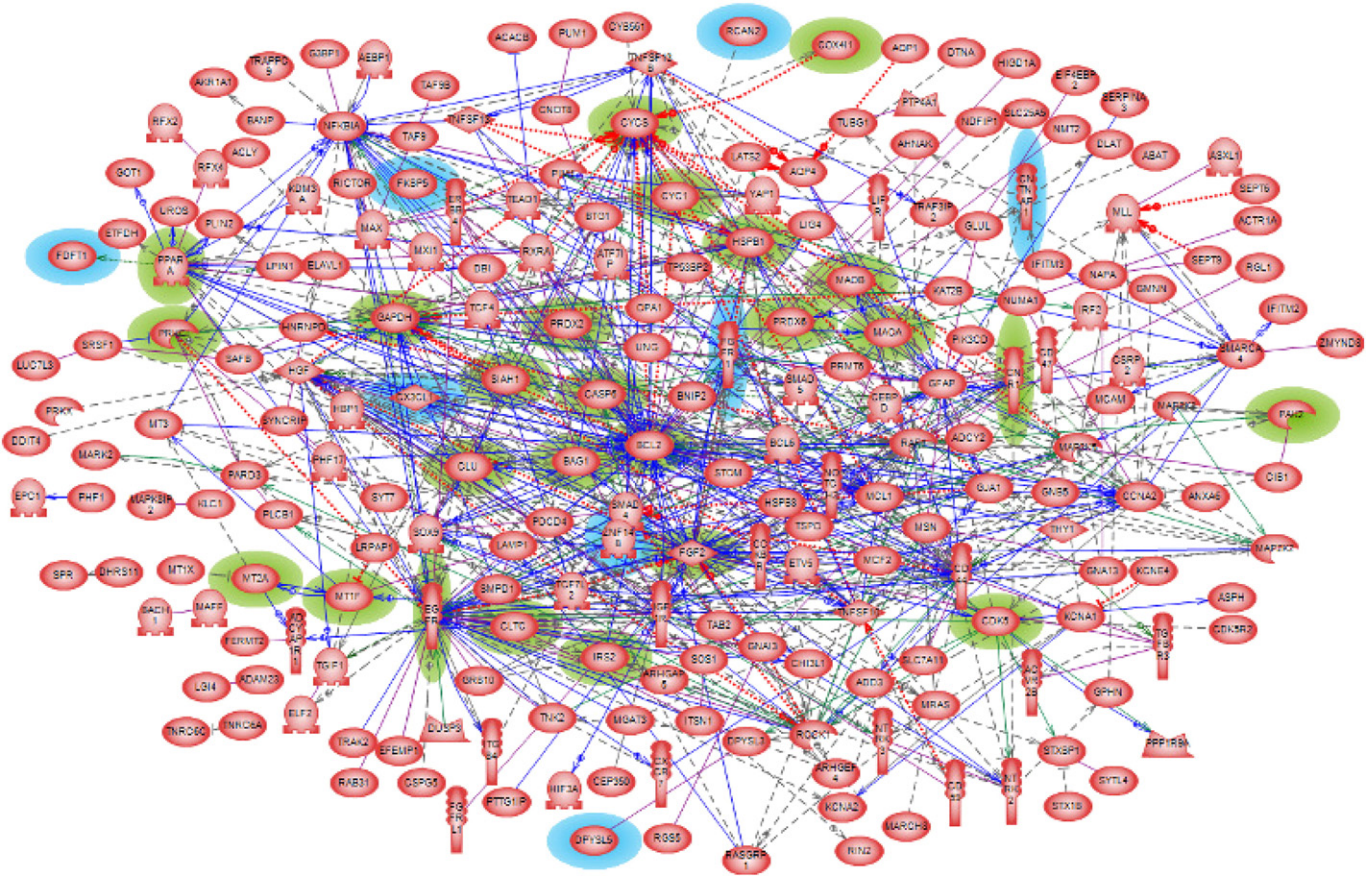
Huntington's disease *direct* interaction network. The 26 genes/proteins implicated in HD pathology are highlighted in green and the eight genes/proteins of potential interest for that disease are highlighted in blue.

Constructing networks using larger gene set such as 531 “seed genes” are beneficial in a couple of ways. First, one could have a broader view of the network neighborhood of any gene of interest along with all its complex interactions. Next, one could also shrink the network size in order to have a closer look on the proximity of those nodes that are critical. Fig. 3 shows the primary *direct* interaction (DI) network of the 531 Huntington's disease “seed genes”. In this network, 224 of these genes *directly* interact with each other and it included such types of interactions like regulation, physical binding, co-expression, promoter binding, protein modification, molecular transport and direct regulation.

**Fig. 4:**
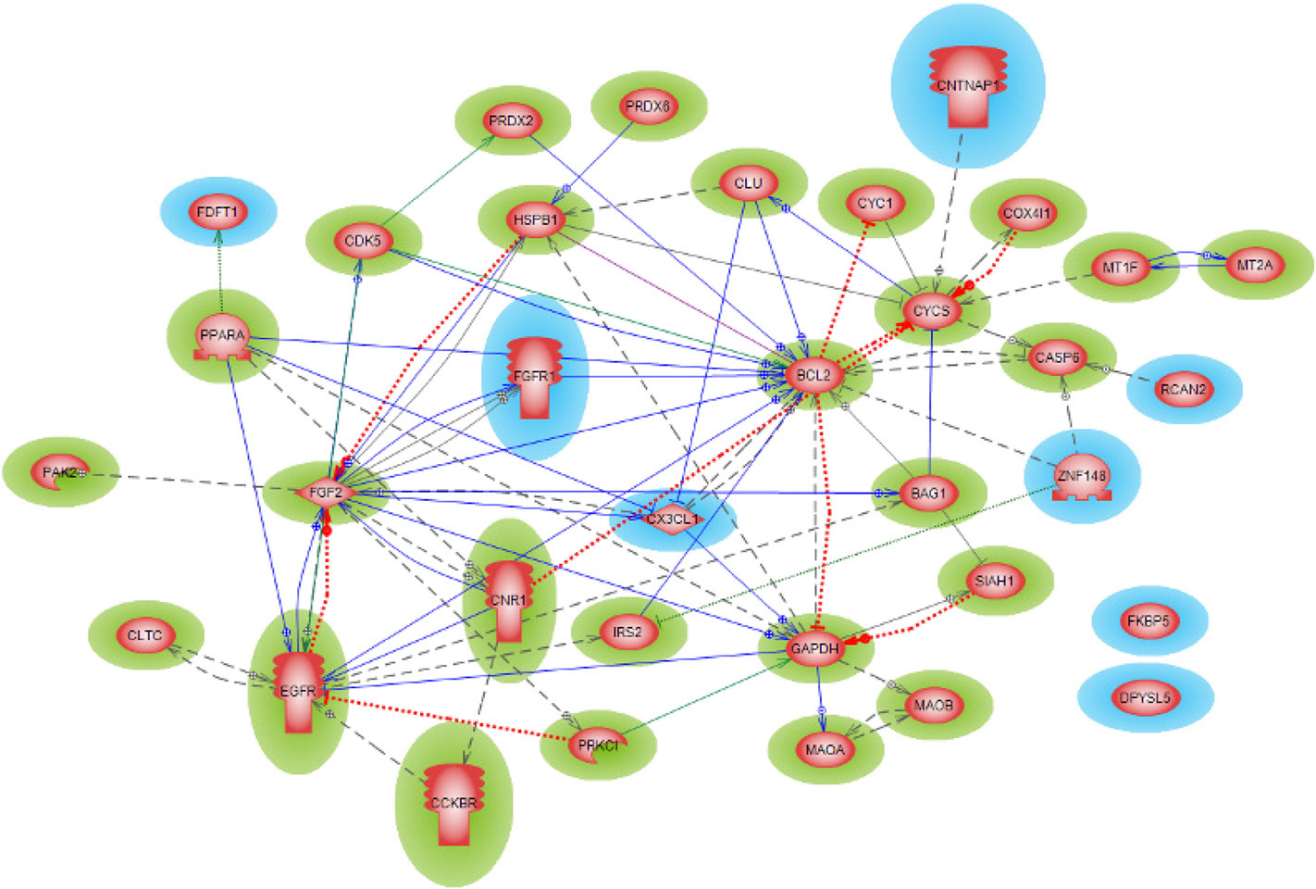
Huntington's disease *compact* direct interaction network. The 26 genes/proteins implicated in HD pathology are highlighted in green and the eight genes/proteins of potential interest for that disease are highlighted in blue.

After exhausted literature review, we found in the direct interaction network 26 genes that have already been associated to Huntington's disease (some of them are discussed in Introduction). Another eight genes (CNTNAP1, CX3CL1, DPYSL5, FDFT1, FGFR1, FKBP5, RCAN2 and ZNF148) were identified as being of potential interest in Huntington's disease pathogenesis due to their specific molecular functions as detailed below. More details on how we conducted our literature search and gene classification are given in methods and data section above, as well as in our Parkinson's paper [36]. We constructed a *compact* direct interaction network using these 34 genes/proteins to understand their inter-connectivity, and to validate the eight genes of interest by the guilt-by-association rule. The network shown in Fig. 4 clearly reveals that except for FKBP5 and DPYSL5, all other genes/proteins of potential interest are connected to the HD-known ones.

Their innate physiological roles along with their vital network attributes, increases the chance of the eight candidate genes to be involved in the HD pathology, elucidating the neuroprotective mechanisms in Huntington's disease realm. For instance, fractalkine (CX3CL1) is a known Parkinson's disease gene where it exhibited neuro protective role against microglia activation as well as reduced motor coordination impairment [50], [51]. Being a known neuroprotective agent for a similar neurodegeneration disease with movement disorder, it may have a potential therapeutic role in Huntington's disease domain too. As mentioned earlier, FGFs were proposed to improve the motor performance and to extend the lifespan in HD mouse model study. Being a receptor for fibroblast growth factors, FGFR1 could up-regulate FGF's beneficial activities in the cell. Studies have found that PPAR-γ together with PGC-1α (a transcriptional co-activator) is required for the regulation of mitochondrial biogenesis. PPAR-γ agonists are thought to be neuroprotective in amyotrophic lateral sclerosis (ALS) and HD [52]. Currently there is no cure available for HD patients. In a search for such a cure, zinc finger proteins were designed in such a way that these proteins were able to recognize and bind specifically with CAG repeats in mouse DNA [53]. The study reported that there was considerable reduction of the mutant huntingtin gene expression at both protein and mRNA levels (95% and 78% reduction, respectively). Many zinc finger proteins including ZBTB10, ZFP36L1 and ZNF148 were found significantly differentially expressed in the microarray dataset used in our study. These zinc finger proteins among which ZNF148 directly interacts with three known-HD genes (BCL2, CASP6 and IRS2) could emerge as a promising new gene therapy tool for Huntington's disease which could be extended and tested in human HD patients.

A number of the candidate genes shown in Fig. 4 are *direct* interacting partners of many already HD-associated genes. The CX3CL1 interacts with five already known-HD genes namely, BCL2, CLU, FGF2, GAPDH and PPARA. The modulation of these previously known-HD genes have been shown beneficial in reducing the disease pathogenesis. This makes this protein a significant player in the HD-related biomolecular mechanisms.

### 3.2 Huntington's disease *shortestpath* network (SPNW)

Different from the previous *direct* interaction network type, *shortest-path* network help us to identify indirect protein–protein interactions that take place through intermediary nodes in the absence of direct relationship. With this in mind, we built the *shortest-path* network using only those seed genes which had at least 25 neighbors in Pathway Studio ResNet 9.0 database (in order to have concise and yet meaningful network). Two hundred fifty-eight out of 531 seed genes met this cut-off criteria and the resulted *shortest-path* network included 208 Pathway Studio software-added connecting genes. Following our methodology, we categorized these connecting genes into two groups. The genes that were already implicated in Huntington's disease belong to the known-HD genes group and those genes which could be of potential interest in HD due to their cellular functions were grouped separately. Table 1 shows the different categories and the number of genes in each.

**Fig. 5:**
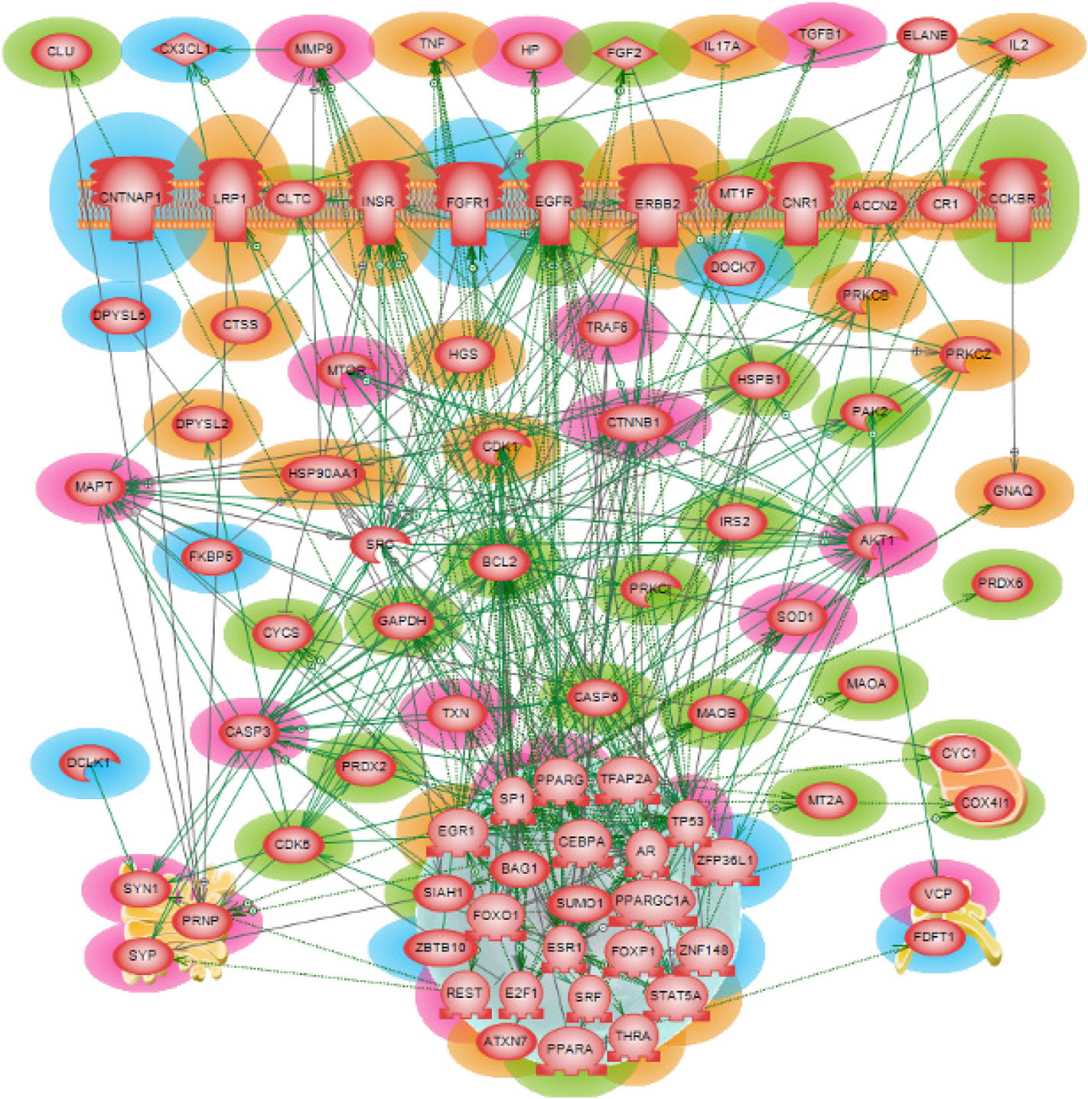
Huntington's disease *compact* shortest path network. The genes/proteins implicated in HD pathology are highlighted in green and red. The genes/proteins of potential interest are highlighted in blue and orange (see Table 1. for details). Genes/proteins causing neuronal loss are highlighted in purple and those that help in neuronal survival are in yellow.

We constructed a *compact* shortest-path network (CSPNW), using the 85 genes from Table 1 along with few additional connecting genes that were needed to have a unified well-connected network. The average node degree of this *compact* shortest-path network was 7.10. In this CSPNW, many of the known-HD genes such as AKT1, AR, BCL2, INSR and SP1 were among the top 25 nodes with node degree ≥ 10, as well as the top 25 with highest closeness (network monitors) and betweenness (traffic influential) centrality measures. Fig. 5 illustrates the interactions between the known and the genes of interest in the HD *compact* SPNW.

Interestingly, PRNP gene/protein was not statistically differentially expressed in our microarray dataset, but it emerged as connecting gene/protein in the *shortest-path* network. PRNP (prion protein) is a glycoprotein that tends to aggregate into rod-like structures causing neuronal cell death. Prion proteins have been associated with many neurodegenerative disorders including Huntington's, Creutzfeldt–Jakob diseases in human, and “mad cow” disease in cattle [54], [55], [56]. We found that PRNP was of importance for network topology as one of the top 15 nodes with highest visibility (closeness) and most influence (betweenness) in the *compact* shortest-path network.

In the next few paragraphs, we summarize the innate molecular characteristics of various genes that could be of potential interest in HD pathogenesis, in addition to their “guilt-by-association” relationship to some of the already implicated HD genes. Table 2 lists our proposed Huntington's disease candidate genes along with the number of known-HD genes to which they directly interact with (see Fig. 5).

From the *compact* shortest-path network, we found that EGR1 (early growth response 1) was the nearest interacting partner to an unusually high number (13) of previously known HD genes (AKT1, AR, BCL2, CLU, CTNNB1, CYCS, FGF2, MAOB, MMP9, SOD1, SP1, TGFB1 and TP53), which according to the “guilt-of-association” rule makes it gene of considerable interest for the HD-disease mechanisms. BIM (BCL2-like 11 apoptosis facilitator) plays an important role in neuronal apoptosis, a hallmark feature of many neurological diseases including Alzheimer's and Parkinson's diseases. Previous research study had demonstrated that EGR1 directly transactivate BIM gene expression to promote neuronal apoptosis. EGR1/BIM pathway has been suggested as a pro-apoptotic mechanism in neurological diseases. Mithramycin A, a U.S. Food and Drug Administration clinically approved drug has been studied to improve motor symptoms and extend life span in a mouse model of Huntington's disease. This drug was suspected to exploit the EGR1/BIM pathway to promote neuroprotective mechanism in HD models and thus could be a promising drug for the treatment for the same [57], [58].

Our next HD candidate gene is CEBPA (CCAAT/enhancer binding protein, alpha). It has been shown to bind to the promoter and modulate the expression of the gene encoding for leptin, a protein that plays an important role in body weight homeostasis. Leptin receptors are found in various brain regions such as the hippocampus and cerebral cortex, and have known roles in neural development and neuroendocrine functions. Studies have indicated that leptin could be neuroprotective and thus enhance neuronal survival [59]. CEBPA, the promoter of leptin gene could also play a critical role in this neuroprotective mechanism. In the *compact* shortest-path network, CEBPA was the first-level interacting partners with nine known-HD genes.

Another recommendation for HD candidate gene is CDK1 (cyclin-dependent kinase 1).The abnormal activation of CDK1 is likely to be involved in the neuronal cell loss in neurodegenerative diseases including Alzheimer's disease and HIV [60]. Earlier, CDK5 was suspected to contribute to the deleterious protein accumulation in Alzheimer's disease [61], [62], [63]. In the *compact* SP network, CDK1 directly interacts with eight known-HD genes. CDK1 is a part of the kinase family that is actively contributing to the neurodegeneration process in similar disease conditions; it could have potential role in HD neurodegeneration mechanism too.

Thus, undeniably, these candidate genes should be further investigated for their molecular role in HD. However, we expect many of these novel genes to surpass the experimental verification due to their “guilt-by-association” with previously established Huntington's disease genes.

Apart from novel connecting genes, it is valuable to note that genes like EGFR, ESR1, HSBP1 and MAPT were also included in the *compact* SP network as connecting genes. They are previously known contributors as well as therapeutic agents in neurodegenerative disorders. Hyperphosphorylated tau (MAPT) is the major component of the neurofibrillary tangles, one of the hallmarks of neurodegenerative diseases [62], [64], [65], [66]. Similarly, huntingtin gene, which mutates strongly in HD was suggested to be indirectly associated with EGFR, thus deregulating the downstream actions of EGFR leading to cell death [67]. Considering the treatment measures, HSBP1 and ESR1 are suggested to offer such mechanisms. In general, heat shock proteins (HSBP1) are evaluated as therapeutic targets in mitigating or preventing protein aggregate formations [68]. One of the major conclusions of an animal HD model study was that the female sex hormone, estrogen (ESR1) could be a target for neuroprotective therapy aiming at postponing the onset and reducing the severity of HD. A similar pattern of late onset was also shown in a human HD study [69], [70]. Moreover, these four connecting genes were among the top 25 nodes with highest connectivity (degree > 10) as well as one of the top 25 nodes with highest visibility as measured by the closeness centrality scores. Except HSBP1, the other three genes were also among the top 25 nodes with highest accessibility to other nodes in the network as determined by the betweenness centrality in the *compact* SP network.

Neueder and Bates [32] employed weighted correlation network analysis to a number of post-mortem brain tissues in human, as well as in mouse samples. They identified extensive transcriptional dysregulation in the cerebellum of HD patients, similar to that observed in the frontal cortex and caudate nucleus. A common signature of gene expression changes in all three brain tissues networks of HD patients has been proposed. Our study generally confirms the disruption of a number of biological processes and first of all that of mitochondrial functions. However, our unweighted correlation network analysis did not show HD importance of some of the genes proposed in their work, e.g., E2F, NRF1, SF1, E4F1, and ELK1 were excluded from our “seed genes” list, due to insufficient statistical significance (*p* > 0.05).

Kalathur et al. [33] used the same GSE3970 database, along with a couple of other HD mouse model datasets. There is a considerable overlap of the enriched KEGG pathways and GO categories with our study. 20 out of 26 Kalahari's selected genes were significantly expressed in our analysis (*p* < 0.05), all of them expressed in caudate nucleus region of the brain. However, due to the more stringent *p*-value cut off criterion in our work (*p* < 0.01), only one gene (CDK5R2) out of these 20 was included in our network analysis. We added also MAP3K5 (*p* < 0.05), as the only gene with more than 25 neighbors.

There is no overlap of genes or biological pathways reported in the study of Alcaraz et al. [34] with those identified in our work. Out of the 11 new genes proposed by these authors seven genes (CTNNB1, GNAQ, GRB2, OPTN, TP53, UBE2K and YWHAB) have *p*-value of < 0.01 but were not significantly expressed in all tissue types so they were not included into our 531 SDEGs.

Continuing with our network analysis, we subjected the genes in the *compact* shortest-path network to DAVID analysis to identify various enriched biological processes and pathways in Huntington's disease. Table 3 lists some of the Gene Ontology categories/subcategories related to nervous system and functions that were statistically significantly enriched in HD (with Benjamini-Hochberg multiple correction). DAVID analysis uncovered biological processes involving in oxidative stress, reactive oxygen species, deregulation in inflammatory response, steroid hormone receptor signaling, lipid binding and insulin receptor signaling pathways to be significantly affected in Huntington's disease, some of which were mentioned above. Other neurodegenerative signaling pathway including Alzheimer's and ALS were also considerably affected in Huntington's disease which reinforce our view for similar underlying molecular pattern in all these diseases. In the next section, we will provide detailed information about our proposed model for Huntington's disease mechanism based on the various biological pathways that were affected in this disease.

### 3.3 Integrated huntington's disease mechanism

In addition to various biological processes, DAVID analysis also recommended several KEGG pathways to be significantly (*p*-values of < 0.05 with Benjamini-Hochberg multiple correction) affected in Huntington's disease of which we selected 10 pathways for further evaluation. These pathways were selected (listed in Table 4) on the basis of previous implications in Huntington's disease research work. Either the entire pathway or many important players of the pathways were found deregulated in HD pathogenesis [71], [72], [73], [74], [75], [76], [77].

**Fig. 6:**
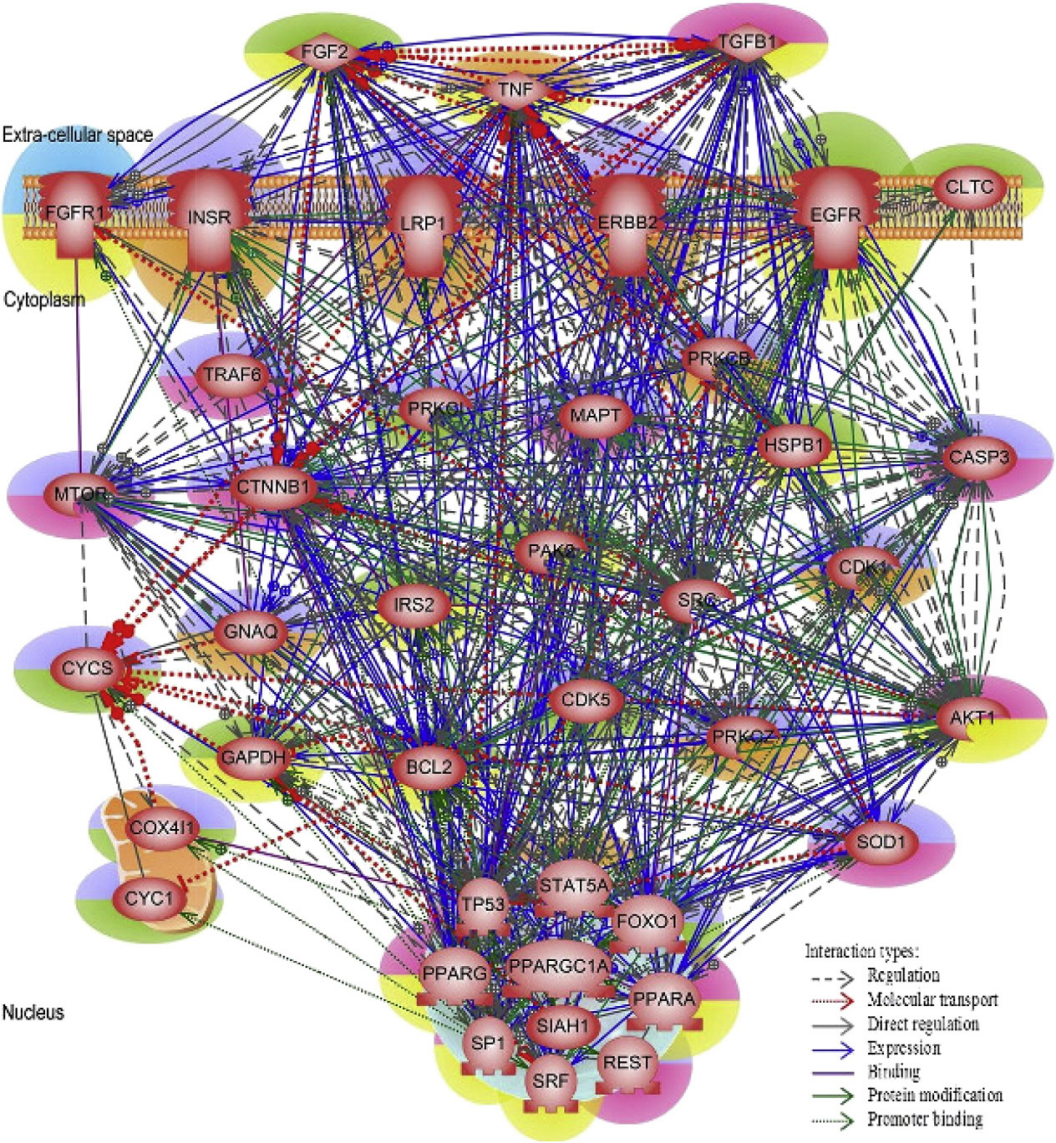
*Integrated* Huntington's disease mechanism. The 41 genes/proteins are found in common in all 10 enriched KEGG pathways. Genes/proteins implicated in HD pathology are highlighted in green/red and the genes/proteins of potential interest are highlighted in blue/orange. Genes/proteins causing neuronal loss are highlighted in purple and those that help in neuronal survival are in yellow.

Enriched KEGG pathways belong to endocrine system, cell communication, cell growth and death, signal transduction, neurodegenerative diseases, and endocrine and metabolic diseases classification. We used the 10 pathways (see Table 4) to search for any underlying molecular mechanism that could either cause or mitigate Huntington's disease pathology. To accomplish this task, we constructed an *integrated* HD mechanism network using the 41 genes found in common in all the 10 enriched KEGG pathways (see Fig. 6).

We then performed a Huntington's disease literature search to classify these 41 genes into two groups, namely, genes that aid in the neuronal survival or cause loss. Twenty-three out of 41 were implicated in neuronal loss and the remaining 18 genes were related to neuronal survival. This classification is depicted in Fig. 6 where genes are highlighted in purple and yellow, respectively. Similar to our *integrated* neurodegenerative disease mechanism networks of Parkinson's and Alzheimer's disease [36], [37], we found a pattern of three extra-cellular ligands (FGF2, TNF, and TGFB1) initiating various downstream signaling cascades in the integrated Huntington's disease mechanism network as well.

As explained earlier, FGF2s are pursued as promising drug targets for its neuroprotective and neuroproliferative roles. TNF (tumor necrosis factor) and TGFB1 (transforming growth factor, beta 1) belong to inflammatory cytokine family which is involved in the regulation of a wide variety of biological processes including cell proliferation, differentiation, adhesion, apoptosis, lipid metabolism, and coagulation. Neuroinflammation has been implicated in many neurological disorders including Huntington's disease. In general, cytokines are required for normal functioning of cells. However, the formation of protein aggregates inside the cell triggers inflammatory mechanism which leads to increased cytokine activities thereby causing chronic cell stress [78], [79]. A delicate balance has to be maintained in order to sustain homeostasis within cell. On examining the *integrated* network, we propose that the hemostasis in Huntington's disease environment could be restored by regulating the three extra-cellular ligands FGF2, TGFB1 and TNF thereby controlling their downstream signaling cascades of various target genes expression. On the other hand, even though the *integrated* HD mechanism network size was relatively small, due to the high interconnectedness of all the nodes it was difficult to suggest specific intracellular pathway(s) to implement in detail the proposed strategy for neuronal restoration in Huntington's disease via the three ligands.

Once again we went back to HD research literature looking for some molecular mechanisms and/or therapeutic pathways that are currently being utilized in this field. A recent review article by Zuccato et al. in 2010 [8] described the past achievements, the current status along with suspected disease mechanisms and therapeutic measures available in Huntington's disease realm. Several research works suggest few important players in HD whose regulation could promote neurogenesis. Under normal physiological conditions, HTT interacts with many genes/proteins including BDNF, MTOR and REST to promote the survival of striatal neurons, the ones that are subjected to cell death in Huntington's disease. Interaction between BDNF (brain-derived neurotrophic factor) and HTT is important for the survival of striatal neurons as well as promoting synapse formation. In addition, HTT binds and sequesters mechanistic target of rapamycin (MTOR) inside the cytoplasm inhibiting MTOR's downstream regulation. In general, MTORs are negative regulators of autophagy. Autophagy is an essential, homeostatic process by which cells break down their own components. They are the debris clearance machineries in the cell that is required to protect against infections, autoimmune and inflammatory diseases [80]. Likewise, REST (RE1-silencing transcription factor) and HTT interaction is also important in HD pathogenesis. HTT binds with REST to maintain low levels of REST gene expression inside the cytoplasm thereby not affecting the transcription of BDNF gene.

In Huntington's disease, mutation in HTT causes protein aggregation formations which were not properly cleared from the cell thus disrupting the normal functioning of the stratial neurons. Due to transcription suppression by REST, the BDNF level was found reduced in neurodegenerative diseases including Alzheimer's, Parkinson's and Huntington's diseases [81], [82]. Mutant HTTs were found inducing neuronal death via distinct but complementary pathways including deregulation of apoptosis and/or autophagy, altered transcription, metabolism and cellular stress responses. Currently one of the therapeutic measures suggested in Huntington's disease domain is clearing the HTT protein aggregates from the cell through the induction of autophagy by the MTOR inhibitor rapamycin. Another indicated treatment is through increasing the beneficial BDNF gene expression [8]. Animal HD model studies have shown that the use of rapamycin (MTOR inhibitor) improved striatal neuron survival and motor performance. However, due to deleterious side effects of rapamycin, it was not recommended for use as an exclusive drug in HD treatment. A combinatorial strategy with rapamycin or other drugs promoting autophagy has been suggested as relevant treatment for HD and other related diseases [83].

**Fig. 7:**
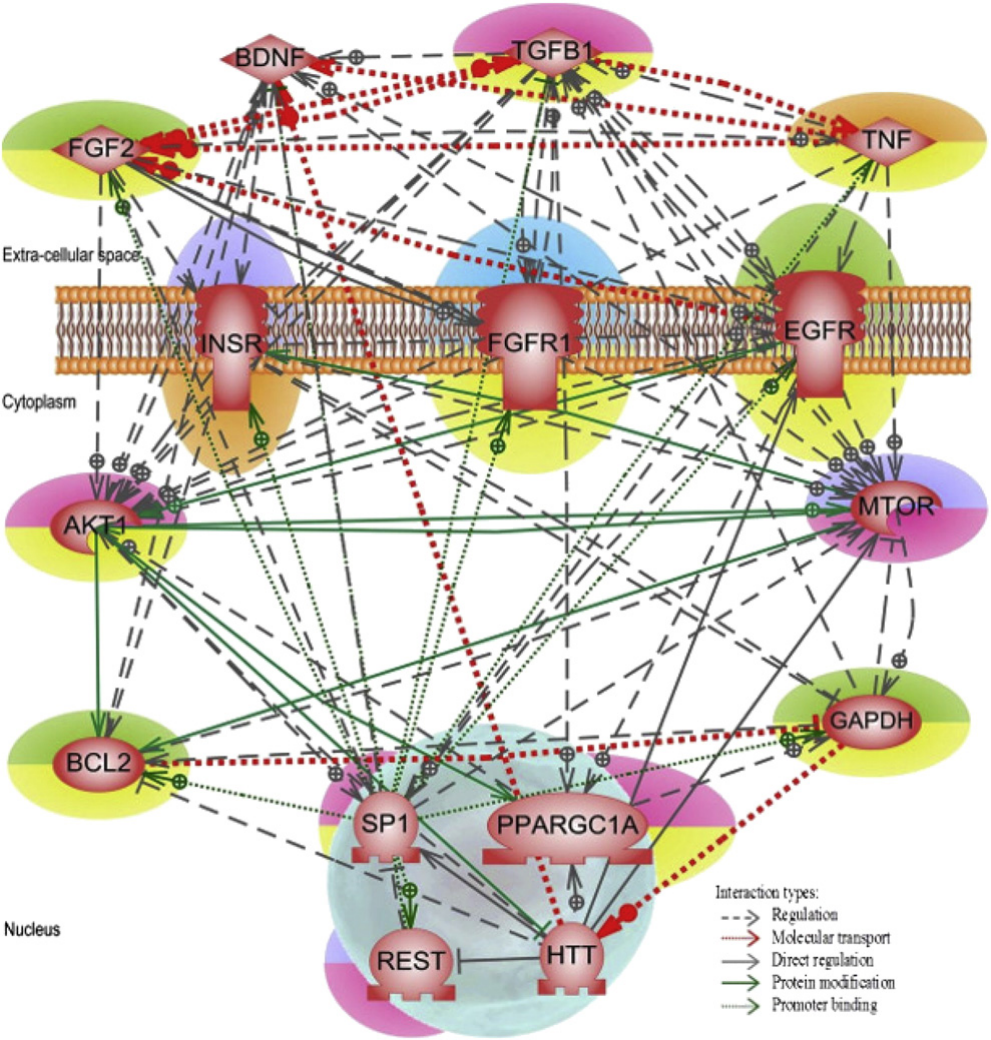
Enriched integrated Huntington's disease mechanism. The 15 genes/proteins that were suggested to play a major role in HD treatment. Genes/proteins implicated in HD pathology are highlighted in green/red and the genes/proteins of potential interest are highlighted in blue/orange. Genes/proteins causing neuronal loss are highlighted in purple and those that help in neuronal survival are in yellow.

Following these HD literature suggested treatment ideas, we modified our *integrated* Huntington's disease mechanism network to include only those nodes (13 genes: AKT1, BCL2, GAPDH, EGFR, FGF2, FGFR1, INSR, MTOR, PPARGC1A, REST, SP1, TGFB1 and TNF) that might play a critical role in both inhibiting MTOR and improving BDNF gene expression. BDNF was added to the reduced network, as was done with HTT. The resulted *enriched* integrated HD mechanism network is shown in Fig. 7.

From this *enriched* network we propose two pathways through which homeostasis in HD could be restored by initiating the downstream signaling cascade of various target genes expression via primarily through TGFB1, one of the three extra-cellular ligands. Our first proposal includes a two-step process of MTOR inhibition. Step 1: TGFB1 activates PPARGC1A gene expression in the nucleus, which in turn increases GAPDH gene/protein activity. Step 2: Up-regulation of GAPDH inhibits MTOR gene expression activity. Once MTOR is inhibited, autophagy mechanism could be boosted up in the cell. As soon as autophagy process is reestablished, HTT protein aggregates will be effectively cleared from the cell thus leading to neuronal survival.

Our second restoration pathway recommendation is via both FGF2 and TGFB1 ligand activation of EGFR receptors thereby initiating several downstream target genes expression. Among those upregulated genes, AKT1 (v-akt murine thymoma viral oncogene homolog 1) is a vital downstream target for EGFR and has been shown to be a critical mediator of neuronal survival. AKT1 has been suggested to be a promising therapeutic target to promote cell survival [84]. Apart from AKT1, EGFR interacts with SP1 (Sp1 transcription factor) which is involved in many cellular processes, including cell differentiation, cell growth, apoptosis, immune responses, response to DNA damage, and chromatin remodeling. SP1 fine-tunes the transcription of many genes including BCL2 and REST. BDNF transcription could be increased via maintaining tight regulation between REST and SP1. In addition, SP1 could also up-regulate BCL2 gene expression, promoting anti-apoptosis. Thus, eventually striatal neurons could be protected by promoting BDNF activity, as well as by reducing the apoptotic process in the cell. Additionally, EGFR also promotes GAPDH gene expression eventually aiding in neuronal survival as detailed in our earlier pathway proposal.

From network analysis stand-point, the two proposed homeostasis restoration pathways show promising measures towards treatment plans in Huntington's disease. As a first step towards translating our proposed therapeutic networks into real world applications, such complex multi-player interconnected pathways could be evaluated using advanced dynamic modeling tools such as cellular automata [85].

### 3.4 Huntington's disease *microRNA* regulatory network

MicroRNAs perform important role in delivering post-transcriptional regulation of gene expression. Previous studies have found such microRNAs in this disease paradigm [86], [87], [88]. In order to identify the microRNAs and their potential targets in Huntington's disease domain, we constructed a *microRNA* regulatory network (MRN) using the 514 “seed genes”. Before proceeding with the MRN construction, we first identified the microRNAs that could target our seed genes. This was accomplished using the *shortest-path* network option in Pathway Studio software where we subjected all the 514 HD “seed genes” to only microRNA interactions type. We found 132 microRNAs to target our HD genes. In order to obtain the microRNA–target gene interactions, we constructed a direct interaction network using the 132 microRNAs and the 514 “seed genes”. [Note: The figure with the microRNA regulatory network containing over 1000 nodes is not shown, due to its extreme complexity].

The average node degree of the microRNA regulatory network was 4.3. Being the node with highest degree in the network, miR-9 was observed to target 35 genes. In addition, miR-9 was the node with highest closeness and betweenness centrality scores. Finding it in our microRNA regulatory network was exciting for a couple of reasons. First, miR-9 was previously known in Huntington's disease mechanism, as well as found to target REST (RE1-silencing transcription factor), one of the important players of HD pathogenesis. Secondly, miR-9 regulation has already been identified and found reduced in both Alzheimer's and Huntington's disease brains [81], [82], [89], [90], providing thus another evidence for the conjectured unified underlined mechanism of the neurodegenerative diseases. Apart from miR-9 regulation, the network included miR-132 and miR-29a/b1 miRNAs, both already associated with HD pathogenesis [91], [92].

The next top five microRNAs found in the regulatory network were miR-124, miR-135a, miR-141, miR-182 and miR-19a. All these microRNAs were among the top 25 nodes with highest degree (node degree ≥ 12), and the top 25 nodes with highest closeness and betweenness centrality scores. miR-124 is one of the most abundantly expressed miRNAs in the nervous system, being widely expressed in neurons in the brain, retina, and spinal cord. It has been implicated in the modulation of neurite outgrowth, as well as cytoskeleton formation [93]. There have been no indications so far for involvement in neurodegenerative processes of miR-135a, known to target genes involved in blood pressure regulation [94]. Similarly, miR-141 and miR-182 were known to be involved only in DNA methylation and cancer metastasis, respectively [95], [96]. miR-19 and miR-21 have been found to target PTEN, a gene/protein found localized in the neurofibrillary tangles (NFTs) and senile plaques in Alzheimer's disease brains [97], [98]. MicroRNA regulatory network also uncovered that many of these top regulating microRNAs modulate several known-HD genes such as CNR1, FOXP1, GAPDH, and IRS2. We suggest that the following nine microRNAs namely, miR-135 A1, miR-141, miR-153-1, miR-15 A, miR-16-1, miR-182, miR-19 A, miR-27 A and miR-96 could be of potential interest in HD. However, our *microRNA* regulatory analysis should be offered with some caution, because miRNAs that are enriched in the CNS are more likely to regulate targets enriched in the CNS. In addition, currently a high percentage of miRNA–target interactions in Pathway Studio ResNet 9.0 database are based on predictions, as verified from the references given in this database. Hence, further experimental verification is recommended.

Table 5 shows the genes of interest in the HD microRNA regulatory network and the number of microRNAs that are targeting each gene. In this network, DOCK7 (dedicator of cytokinesis 7) was the gene with the highest number of microRNA regulations, being regulated by seven members of miR-181 and miR-30 families. DOCK7 gene encodes for guanine nucleotide exchange factor (GEF) protein that plays a major role in axon formation and neuronal polarization. In general, GEFs are critical mediators of Rho GTPase activation by stimulating the exchange of GDP for GTP. Under normal physiological conditions, Rho GTPases act as molecular switches in intracellular signaling pathways and have many downstream targets. Mutations in GEFs and deregulated Rho GTPase signaling have been implicated in ALS, a debilitating motor neuron disease caused by neuronal degeneration. Based on its molecular function and its association with similar neurodegeneration disease, DOCK7 could be of potential interest in Huntington's disease mechanism as well.

Moving on with other genes of interest in the microRNA regulatory network, zinc finger proteins (ZBTB10, ZNF148, and ZFP36L1) were highly targeted by multiple microRNAs including miR-20a and miR-29b1, known microRNAs in Alzheimer's and Huntington's diseases, respectively [99]. As reported in the previous section, zinc finger proteins are demonstrated to be a promising new gene therapy tool for Huntington's disease. Such a therapy could be enhanced by these microRNA regulations.

Additional to *microRNA* regulation, the network also included 43 genes that code for transcription factors. These significantly differentially expressed TFs indicate a possible integrated gene expression regulation mechanism in Huntington's disease. Like we noticed for the other two neurodegenerative disorders, there is a likelihood of dual-level gene expression regulation also occurring in HD paradigm. Thus, Huntington's disease should be considered as another complex disease system that involves highly interconnected molecular players and multi-level regulation.

## 4 Conclusions

Proceeding from 514 well selected “seed genes” that were significantly differentially expressed in HD postmortem samples we constructed several types of intracellular networks. Our network analysis was based on two basic principles. The guilt-by-association rule gives preference to genes being surrounded in the network predominantly by genes implicated with Huntington's disease. The best example of this strategy is our candidate gene *EGR*1 which interacts with 13 already known HD genes. Our second prioritizing rule was based on identifying *critical nodes in network topology*, i.e., nodes with high connectivity, visibility and traffic influence, as characterized by the node degree, closeness centrality and betweenness centrality. Thus, five of our novel candidate genes: *EGR1*, *CDK1*, *CEBPA*, *E2F1*and *INSR* were among the top 25 most highly connected, visible and influential genes. Moreover, some well-known Huntington's disease genes like *PRNP* which have not been significantly expressed in the post-mortem microarrays and was not included in our “seed gene” list, re-emerged as one of the top 15 critical nodes with highest visibility and most influence on the interaction traffic.

Overall, our network analysis prioritized 19 novel genes and nine miRNAs with pivotal positions in the HD-related networks built. Taking also in consideration their intrinsic molecular functions, we propose these genes and miRNAs as novel candidates for the analysis of Huntington's disease pathogenesis and survival, and briefly discuss some of those. *CEBPA* was among the best connected with known-HD genes. As promoter of leptin gene which enhances neuronal survival CEBPA could also play a critical role in the neuroprotective mechanism. Another well-connected gene is *CDK*1, a part of the kinase family that is actively contributing to the neurodegeneration process in similar disease conditions. The early growth response gene 1 (*EGR1*), which plays a role in memory formation, has a record-high connectivity to 13 HD-related genes and may also be considered as strong candidate for experimental confirmation of its role in the Huntington's disease. *E2F1* mediates neuronal death via activation of its transcriptional targets. *ERBB2* is of interest as a link between molecular pathways underlying neurodegeneration. *LRP1* seems to be of importance in elucidating the connection between cholesterol homeostasis and pathophysiology of HD. *HSP90AA*1 as a member of heat shock proteins, might be critically involved in the progression of HD. As a member of the zinc finger proteins family *ZNF*148 could be of interest in regulating the level of the mutant Huntingtin protein. Their critical role in the biological pathways that was significantly affected in Huntington's disease, as well as being directly associated with many known-HD genes, increases the probability that these proposed candidate genes could play a major part in the HD pathogenesis.

Through our *microRNA* regulatory network, we suggest that nine microRNAs could be potential regulators and drug targets in HD. However, the central role in our network is played by two well-known ones, miR9 and miR124. We plan to investigate the possibility for dual-level gene regulation by both microRNAs and transcription factors in Huntington's disease mechanism. Our future work on the role of miRNAs in HD pathogenesis and molecular mechanisms for fighting the disease will account for the limitations of the microarray analysis. The latter is capable of measuring the status of known transcripts only, and expression of low-abundance mRNAs is often not detected by the hybridization-based approach, thus opening the field for the more sensitive RNA-seq analysis, a revolutionary tool for transcriptomics and neurodegenerative diseases [100], [101].

Analyzing our *integrated* network, we propose plans for several beneficial pathways of modulations of HD-related molecular factors, initiated via the extra-cellular ligands TGFB1, FGF2 and TNF. Restoring the normal homeostasis in Huntington's disease seems possible; one such plan is to up-regulate the innate autophagy process by inhibiting MTOR activity within the cell. Another plan aims to promote striatal neuron survival via increasing BDNF gene expression.

The following are the supplementary data related to this article.

Table S1 Four-set Venn diagram of the overlap of significantly differentially expressed genes (SDEGs).

## Figures and Tables

**Table 1:** Summary of genes of interest and genes already known in Huntington's disease.

Different categories	No. of genes	Node color code in figures
Genes of interest from SDEGs	13	blue
Known HD genes from SDEGs	25	green
Genes of interest in SPNW connecting nodes	23	orange
Known HD genes in SPNW connecting nodes	24	red

**Table 2:** Genes of interest for Huntington's disease identified by “guilt-by-association” with the known HD-related genes.

Genes of interest	Interacts with no. of known HD genes
EGR1	13
CEBPA	9
CDK1	8
HSP90AA1	7
PRKCZ	6
E2F1, STAT5A	5
SRF	4
ERBB2, FGFR1, IL2, INSR, LRP1, PRKCB, TNF, ZNF148	3
CX3CL1	2
CNTNAP1, RCAN2	1

**Table 3:** Gene set DAVID enrichment analysis of Huntington's disease *compact* shortest-path network.

Term	Gene count	Fold enrichment	Benjamini
GO:0048666 ~ neuron development	14	6.50	5.99E − 06
GO:0030182 ~ neuron differentiation	15	5.39	1.44E − 05
GO:0046324 ~ regulation of glucose import	6	28.60	4.33E − 05
GO:0043005 ~ neuron projection	13	6.07	1.37E − 04
GO:0050994 ~ regulation of lipid catabolic process	5	30.25	3.09E − 04
GO:0010001 ~ glial cell differentiation	6	17.81	3.09E − 04
GO:0043523 ~ regulation of neuron apoptosis	7	12.23	3.22E − 04
GO:0031175 ~ neuron projection development	10	6.14	4.40E − 04
GO:0043627 ~ response to estrogen stimulus	7	10.49	6.42E − 04
GO:0042063 ~ gliogenesis	6	14.52	6.76E − 04
GO:0048812 ~ neuron projection morphogenesis	9	6.65	6.99E − 04
GO:0006979 ~ response to oxidative stress	8	7.67	8.94E − 04
GO:0001836 ~ release of cytochrome c from mitochondria	4	29.96	0.003
GO:0048667 ~ cell morphogenesis involved in neuron differentiation	8	6.02	0.003
GO:0030425 ~ dendrite	8	7.84	0.004
GO:0030424 ~ axon	8	8.04	0.004
GO:0007568 ~ aging	6	8.58	0.006
GO:0008289 ~ lipid binding	11	3.82	0.009
GO:0000302 ~ response to reactive oxygen species	5	10.49	0.010
GO:0050727 ~ regulation of inflammatory response	5	10.35	0.011
GO:0007409 ~ axonogenesis	7	5.71	0.011
GO:0008286 ~ insulin receptor signaling pathway	4	17.01	0.013
GO:0050804 ~ regulation of synaptic transmission	6	6.94	0.013
GO:0031644 ~ regulation of neurological system process	6	6.17	0.020
GO:0016192 ~ vesicle-mediated transport	11	3.00	0.023
GO:0050767 ~ regulation of neurogenesis	6	5.69	0.027
GO:0030518 ~ steroid hormone receptor signaling pathway	4	10.85	0.037
GO:0006874 ~ cellular calcium ion homeostasis	6	5.16	0.038
GO:0030136 ~ clathrin-coated vesicle	6	7.26	0.039
GO:0055114 ~ oxidation reduction	11	2.71	0.042
GO:0045121 ~ membrane raft	6	6.70	0.045

**Table 4:** Enriched KEGG pathways in Huntington's disease resulted from DAVID analysis.

Term	Gene count	Fold enrichment	Benjamini	Genes
hsa05016:Huntington's disease	12	5.14	4.40E − 04	CASP3, GNAQ, SP1, CYCS, PPARG, CYC1, TP53, COX4I1, REST, CLTC, SOD1, PPARGC1A
hsa04010:MAPK signaling pathway	14	4.04	5.86E − 04	EGFR, FGFR1, TNF, TP53, SRF, TGFB1, PRKCB, AKT1, CASP3, PAK2, MAPT, HSPB1, TRAF6, FGF2
hsa04012:ErbB signaling pathway	8	7.08	0.002	EGFR, AKT1, PAK2, ERBB2, STAT5A, MTOR, SRC, PRKCB
hsa05010:Alzheimer's disease	10	4.73	0.003	CASP3, TNF, LRP1, GNAQ, MAPT, CYCS, CYC1, COX4I1, GAPDH, CDK5
hsa05014:Amyotrophic lateral sclerosis (ALS)	6	8.72	0.006	CASP3, TNF, BCL2, CYCS, TP53, SOD1
hsa04910:Insulin signaling pathway	8	4.57	0.011	AKT1, PRKCZ, IRS2, PRKCI, FOXO1, MTOR, INSR, PPARGC1A
hsa04920:Adipocytokine signaling pathway	6	6.90	0.012	AKT1, PPARA, IRS2, TNF, MTOR, PPARGC1A
hsa04930:Type II diabetes mellitus	5	8.20	0.015	PRKCZ, IRS2, TNF, MTOR, INSR
hsa04520:Adherens junction	6	6.00	0.016	EGFR, FGFR1, ERBB2, INSR, SRC, CTNNB1
hsa04115:p53 signaling pathway	5	5.67	0.049	CDK1, CASP3, CYCS, TP53, SIAH1

**Table 5:** Genes of interest determined from Huntington's disease microRNA regulatory network.

Genes of interest	Target by no. of miRNAs
DOCK7, ZBTB10	7
DPYSL5, ZNF148	6
DCLK1, OSBPL11, RCAN2, ZFP36L1	5
CNTNAP1	4
FGFR1, FKBP5	2
CX3CL1, FDFT1	1
